# Activation of ETAR and ETBR in myocardial tissue characterizes heart failure induced by experimental autoimmune myocarditis

**DOI:** 10.1186/s12872-023-03658-1

**Published:** 2024-01-02

**Authors:** Peng Yang, Yujing Wu, Fangfei Li, Jiangfeng Tang, Zhenzhong Zheng, Qingshan Tian

**Affiliations:** 1 Department of Cardiology, The First Affiliated Hospital, Jiangxi Medical College, Nanchang University, Nanchang, Jiangxi 330006 China; 2 Department of Cardiology, Shenzhen Third People’s Hospital, Shenzhen, 518112 Guangdong China

**Keywords:** ET-1, ETAR, ETBR, Myocarditis, Heart failure, Endothelial dysfunction

## Abstract

**Background:**

Endothelial dysfunction is characterized by an imbalance between endothelium-derived vasodilatory and vasoconstrictive effects and may play an important role in the development of heart failure. An increasing number of studies have shown that endothelial-derived NO-mediated vasodilation is attenuated in heart failure patients. However, the role of endothelin-1 (ET-1) in heart failure remains controversial due to its different receptors including ET-1 receptor type A (ETAR) and ET-1 receptor type B (ETBR). The aim of this study was to determine whether ET-1 and its receptors are activated and to explore the role of ETAR and ETBR in heart failure induced by myocarditis.

**Methods:**

We constructed an animal model of experimental autoimmune myocarditis (EAM) with porcine cardiac myosin. Twenty rats were randomized to the control group (3 weeks, *n* = 5), the extended control group (8 weeks, *n* = 5), the EAM group (3 weeks, *n* = 5), the extended EAM group (8 weeks, *n* = 5). HE staining was used to detect myocardial inflammatory infiltration and the myocarditis score, Masson’s trichrome staining was used to assess myocardial fibrosis, echocardiography was used to evaluate cardiac function, ELISA was used to detect serum NT-proBNP and ET-1 concentrations, and immunohistochemistry and western blotting were used to detect ETAR and ETBR expression in myocardial tissue of EAM-induced heart failure. Subsequently, a model of myocardial inflammatory injury in vitro was constructed to explore the role of ETAR and ETBR in EAM-induced heart failure.

**Results:**

EAM rats tended to reach peak inflammation after 3 weeks of immunization and developed stable chronic heart failure at 8 weeks after immunization. LVEDd and LVEDs were significantly increased in the EAM group compared to the control group at 3 weeks and 8 weeks after immunization while EF and FS were significantly reduced. Serum NT-proBNP concentrations in EAM (both 3 weeks and 8 weeks) were elevated. Therefore, EAM can induce acute and chronic heart failure due to myocardial inflammatory injury. Serum ET-1 concentration and myocardial ETAR and ETBR protein were significantly increased in EAM-induced heart failure in vivo. Consistent with the results of the experiments in vivo, ETAR and ETBR protein expression levels were significantly increased in the myocardial inflammatory injury model in vitro. Moreover, ETAR gene silencing inhibited inflammatory cytokine TNF-α and IL-1β levels, while ETBR gene silencing improved TNF-α and IL-1β levels.

**Conclusions:**

ET-1, ETAR, and ETBR were activated in both EAM-induced acute heart failure and chronic heart failure. ETAR may positively regulate EAM-induced heart failure by promoting myocardial inflammatory injury, whereas ETBR negatively regulates EAM-induced heart failure by alleviating myocardial inflammatory injury.

**Supplementary Information:**

The online version contains supplementary material available at 10.1186/s12872-023-03658-1.

## Background

Myocarditis is an inflammatory disease of the heart caused by various aetiologies, with viral infections being the most common cause of myocarditis [1]. Approximately 40–66% of patients with myocarditis recover completely on their own within the first 4–12 weeks. However, approximately half of myocarditis patients with myocardial immune damage often develop dilated cardiomyopathy (DCM) and heart failure (HF) later in life, mainly manifested by chronic inflammatory cell infiltration, left ventricular dilatation, septal thinning, and reduced ejection fraction [2–4]. Clinically, myocarditis HF accounts for approximately one in nine cases of nonischemic HF and is a common cause of hospitalization and death [5]. Unfortunately, the mechanism of myocarditis has not been fully elucidated, and no effective treatment strategy for myocarditis is available.

Endothelial cells have endocrine and paracrine activity, and endothelial cells release a variety of diastolic and vasoconstrictive substances that play an important role in the regulation of vascular function and structure [6]. Nitric oxide (NO) is the main strong vasodilator produced by endothelial cells and has an important protective role in maintaining normal cardiovascular function [7]. Endothelial dysfunction is a complex pathological state manifested mainly by decreased nitric oxide production capacity and reduced nitric oxide sensitivity [8]. Endothelial dysfunction is an important initial event at the onset of cardiovascular disease, and is closely related to the occurrence and development of HF [9]. Studies have shown that endothelial-derived NO-mediated vasodilation is attenuated in HF patients, both in HF with reduced ejection fraction (HFrEF) and in HF patients with preserved ejection fraction (HFpEF) [10].

Endothelin-1 (ET-1), an active peptide composed of 21 amino acids, is the main vasoconstrictor secreted by endothelial cells. It antagonizes the vasodilatory effect of NO and has prooxidant and inflammatory properties [11]. However, unlike NO, the role of ET-1 and ET-1 receptors (ETRs) in HF remains controversial. ET-1 mediates its action through two types of receptors, ET-1 receptor type A (ETAR) and ET-1 receptor type B (ETBR). In most arterial smooth muscle cells and some venous smooth muscle cells, ET-1 elicits a vasoconstrictor response by stimulating ETAR on the cell membrane. Conversely, ET-1 stimulates ETBR on endothelial cells and releases NO, which produces vasodilation [12].

Endmyocardial biopsy, the gold standard for the diagnosis of myocarditis, is rarely used due to its invasive nature, which makes the diagnosis of myocarditis largely dependent on the patient's clinical symptoms as well as on clinical biochemical and imaging examinations. Moreover, its relationship with HF has not received sufficient attention. Experimental autoimmune myocarditis is an animal model of human myocarditis and DCM, which is important for elucidating the pathogenesis of the disease and developing immunotherapy [13]. In most studies, cardiac myosin was used as an antigen (Ag) to induce myocarditis. In this paper, we focus on the induction of experimental autoimmune myocarditis (EAM) by porcine cardiac myosin (PCM), explore the relationship between myocarditis and acute and chronic HF, and further investigate the value of the ET-1/ETR axis in acute and chronic HF to improve the clinical diagnosis and treatment strategy of HF.

## Materials and methods

### Animals and experimental study design

Twenty male Lewis rats were purchased from Vital River Laboratory Animal Technology Co. (Beijing, China). At the time of the EAM model test the rats were 8–9 weeks old and had body weights of 212.3 ± 3.7 g. Animals were kept under standard conditions with a mean temperature of 25 °C ± 2 °C, a mean relative humidity of 50% ± 20 and a light–dark cycle of 12:12 h. PCM-induced EAM rats tended to reach peak inflammation after 3 weeks of immunization and develop stable chronic HF at 8 weeks of immunization [14, 15]. Therefore, 10 immunized rats were randomized to the EAM group (3 weeks, *n* = 5) or EAM group (8 weeks, *n* = 5). Ten age-matched normal Lewis rats were used as the corresponding control group. Experimental and control rats in the acute HF group were sacrificed on Day 21 after immunization, while experimental and control rats in the chronic HF group were sacrificed on Day 56 after immunization. No control diet was needed in this experiment, and all diets were standardized. Rats were sacrificed by intravenous injection of an overdose of pentobarbital (100–150 mg/kg). After sacrifice, the heart tissue was separated and rapidly fixed in 4% paraformaldehyde purchased from Beyotime Biotechnology Co. (Shanghai, China).

### EAM animal model induction

The purified PCM (Sigma Aldrich, M0531) was dissolved in 0.15 mol/L phosphate-buffered saline (PBS, Servicebio, G4202), and the final concentration was adjusted to 2 g/L. Then, the PCM was emulsified with Complete Freud’s Adjuvance (CFA, Sigma Aldrich, F5881) in a 1:1 ratio. Each rat in the experimental group was injected subcutaneously with 200 μL PCM-CFA emulsion in the bilateral groin, foot pads, and axillae on Day 1 and Day 7. Rats in the control group were injected subcutaneously with 100 μL of CFA on Day 1 and Day 7.

### Cell cultures

H_9_C_2_ rat cardiomyoblasts were cultured in dulbecco’s modified eagle medium (DMEM) supplemented with 10% fetal bovine serum (FBS, Cellmo, SA301.02) and 1% penicillin–streptomycin (PS, Biotech, C100C5) at 37 °C with 5% CO_2_. After the cells were observed under the microscope to adhere to the wall, the cells were digested with 1 mL of 0.25% trypsin–EDTA (Biotech, C100C1) for passage when the cell density reached 80% to 90%. H_9_C_2_ cells were purchased from Servicebio Technology Co.,Ltd (Hubei, China).

### Construction of the myocardial inflammatory injury model in vitro

An in vitro myocardial inflammatory injury model was constructed according to the studies of Wang, R., et al. [16] and Mirna, M.,et al. [17]. H_9_C_2_ cell suspensions were incubated in a 6-well plate and 2 mL of DMEM containing 10% FBS was added to each well. When the cell density reached 70–80%, H_9_C_2_ cells were stimulated with 10 μg/mL lipopolysaccharides (LPS) and incubated in a 5% CO_2_ incubator at 37℃ for 12 h.

### Cell transfection

For siRNA transfection, H_9_C_2_ cells were seeded in 6-well plates one day in advance. After the cells grew to 50% adherence, 20 nM ETAR or ETBR siRNA was transfected using jetPRIME Transfection Reagent (polyplus, 101,000,046) for 48 h. The protein expression levels of ETAR and ETBR was measured by western blot to determine the best interference effect. The siRNA sequences of the ETAR and ETBR genes were described in Table 1.

**Table 1:** SiRNA sequences of ETAR and ETBR gene

Sequence gene	Sequence (5'to 3')
Si ETAR #1 -F	CUGACAAUGCUGAGAGAUATT
Si ETAR #1 -R	UAUCUCUCAGCAUUGUCAGTT
Si ETAR #2 -F	CGACCAAGUUCAUGGAGUUTT
Si ETAR #2 -R	AACUCCAUGAACUUGGUCGTT
Si ETAR #3 -F	ACCAUGAACUCUUGCAUAATT
Si ETAR #3 -R	UUAUGCAAGAGUUCAUGGUTT
Si ETAR NC -F	UUCUCCGAACGUGUCACGUTT
Si ETAR NC -R	ACGUGACACGUUCGGAGAATT
Si ETBR #1 -F	AGAACAAGUGCAUGAGAAATT
Si ETBR #1 -R	UUUCUCAUGCACUUGUUCUTT
Si ETBR #2 -F	ACUAAGACCUCCUGGACUATT
Si ETBR #2 -R	UAGUCCAGGAGGUCUUAGUTT
Si ETBR #3 -F	CAACAUGGCUUCUUUGAAUTT
Si ETBR #3 -R	AUUCAAAGAAGCCAUGUUGTT
Si ETBR NC-F	UUCUCCGAACGUGUCACGUTT
Si ETBR NC-R	ACGUGACACGUUCGGAGAATT

### Assessment of cardiac function by echocardiography

Transthoracic echocardiography was performed using a Sonos 5500 ultrasound machine (Phillips, USA) with a 12 MHz phased array transducer, real-time digital acquisition, storage, and review capabilities. On Day 21 and Day 56, rats were anaesthetized with 2% pentobarbital sodium (50 mg/kg) by intraperitoneal injection and the chest was shaved. Rats were placed on a shallow left lateral position. Two-dimensional mode, M-mode, Doppler flow images were obtained in parasternal long/short-axis view. The left ventricular end-systolic diameters (LVEDs) and left ventricular end-diastolic diameters (LVEDd) were measured over the course of at least 3 consecutive cardiac cycles. The left ventricular ejection fraction (EF) and short axis shortening fraction (FS) were calculated.

### Histopathological examination

The heart tissue was fixed in 4% paraformaldehyde in PBS and embedded in paraffin wax. Sections were cut 5 µm thick at various depths in the tissue section and stained with haematoxylin and eosin (HE). The central portions of each stained section were examined under a light microscope to determine myocarditis severity. The degree of myocardial infiltration and fibrosis was determined blindly by two independent investigators in a blinded manner. Five high magnification fields were randomly selected under the microscope, the ratio of the area of inflammatory cell infiltration and necrosis in each field to the area of the whole field was calculated, and its mean value was used for the myocarditis score. The myocarditis scores were exactly as follows: 0, no inflammation; 1, < 25% of the heart section involved; 2, 25–50%; 3, 50–75%; and 4, > 75% [18].

### Masson trichrome staining

Myocardium section (5 µm) were stained with Masson Trichrome according to the manufacturer’s instructions for the Trichrome Stain (Masson) Kit (Solarbio, G1340), which was used to assess myocardial tissue fibrosis. At least 3 microscopic fields were randomly selected at × 100 magnification for each section to be photographed. The photographs were taken so that the tissue filled the entire field of view as much as possible, ensuring that the background light was the same for each photograph. Image analysis software (Image-Pro Plus 6.0) was applied to select the same blue colour as a uniform standard for judging collagen fibers in all photographs, and each photograph was analysed to derive the collagen volume fraction (CVF, CVF = myocardial collagen fiber area/total area of the image).

### Enzyme-linked immunosorbent assay (ELISA)

After echocardiography, blood was collected from the inferior vena cava and centrifuged with a speed of 3000 rpm for 10 min, and the rat serum was separated. The serum ET-1 concentration was measured by an ET-1 ELISA kit according to thespecifications of the ELISA kit. The ELISA kit was purchased from Senxiong Technology Industrial Co. (Shanghai, China). The N-terminal pro-B type brain natriuretic peptide (NT-proBNP) concentration in serum was estimated by an NT-proBNP ELISA kit which was purchased from Abcam Trading Co. (Shanghai, China). The supernatant of H_9_C_2_ cells was collected, and according to the manufacturer's instructions, ELISA kits for IL-1β and ELISA kits for TNF-α were used to detect IL-1β and TNF-α concentrations, respectively. ELISA kits of IL-1β and TNF-α were obtained from Meimian Industrial Co.,Ltd (Jiangsu, China).

### Immunohistochemical determination of ETAR and ETBR

To evaluate the expression of ETAR and ETBR, we performed immunohistochemistry. The samples were processed according to standardized protocols in the routine histopathology laboratory. Briefly, after formalin fixation, they were cut into 2 mm slices and embedded in paraffin. Each 5 μm thin slice was mounted on glass slides, deparaffinized in xylene and rehydrated by sequential rinses in absolute, 95%, 80%, and 70% ethanol. Endogenous peroxidase activity was exhausted by incubation with 3% H_2_O_2_. Sections were then washed in PBS, preincubated in 5% bovine serum albumin (BSA) for 30 min and incubated overnight on a shaker at room temperature with rabbit polyclonal anti-endothelin A Receptor antibodies (diluted 1:50) (Abcam, ab178454) rabbit polyclonal anti-endothelin B Receptor antibodies (diluted 1:50) (Abcam, ab117529) directed against ETAR and ETBR. The sections were then washed and incubated in biotinylated anti-rabbit or anti-goat secondary antibody (diluted 1:1500) at 37℃ for 50 min. The signal was amplified with an acidin-biotin-horseradish peroxidase procedure (vector) and visualized with diaminobenzidine as the chromogen. Negative control slides were included in all experiments in which, the antibody was omitted and replaced by control irrelevant diluent. The immunostaining of ETAR and ETBR was performed by greyscale test with a computer-assisted image analysis system (HMIAS-2000, Champion Medical Imaging Co). The same brown colour was selected as a uniform criterion for judging all positive images, and each image was analysed to derive the integrated option density (IOD) for each positive image and the pixel area of the tissue (AREA). The ratio of IOD to AREA (IOD/AREA) was derived for ETAR and ETBR protein relative expression.

### Western blot analysis

For western blot analysis, total proteins were extracted from radioimmunoprecipitation assay (RIPA) lysis buffer (Solarbio, R0020). Protein was quantified by a bicinchoninic acid assay (BCA) protein assay kit (Beyotime, P0012S). Total protein was denatured by boiling for 10 min. Protein (40 µg) was separated by 10% sodium dodecyl sulfate‒polyacrylamide gel electrophoresis (SDS-PAGE) (Bio-Rad, CA, USA) and transferred to a nitrocellulose filter membrane (Millipore, Massachusetts, USA). After blocking with 5% skim milk for 1 h at room temperature, the membranes were incubated with the following primary antibodies: rabbit polyclonal anti-endothelin A Receptor antibodies (Abcam, ab178454, 1:1000 in 5% skim milk), rabbit polyclonal anti-Endothelin B Receptor antibodies (Abcam, ab117529, 1:1000 in 5% skim milk), and rabbit polyclonal anti-GAPDH (Abcam, ab37168, 1:1000 in 5% skim milk) for 12 h to 16 h. Then, peroxidase-conjugated AffiniPure goat anti-rabbitIgG(H + L)(ZSGE-BIO, ZB-2301, 1:10,000 in 5% skim milk) was added for 2 h at room temperature and subsequently developed using an enhanced chemiluminescence reagent (Beyotime, P0018A). The signals emitted for chemiluminescence were detected using the Image Quant LAS 4000 (Bio-Rad, CA, USA, 00746947) and analysed with ImageJ software (NIH). The results were normalized to the corresponding densitometry signal of GAPDH.

### Statistical analysis

All statistical analyses were performed using SPSS version 20.0 software(IBM Corporation,Somers, NY, USA) and GraphPad Prism version 8.0.1 software (GraphPad Software, Inc., CA, USA). Continuous data are presented as the mean ± SEM, and comparisons were performed using two-way ANOVA method. *P* value < 0. 05 was considered statistically significant. *P < 0.05, **P < 0.01).

## Results

### PCM-induced EAM rats’ physiological conditions

**Fig. 1:**
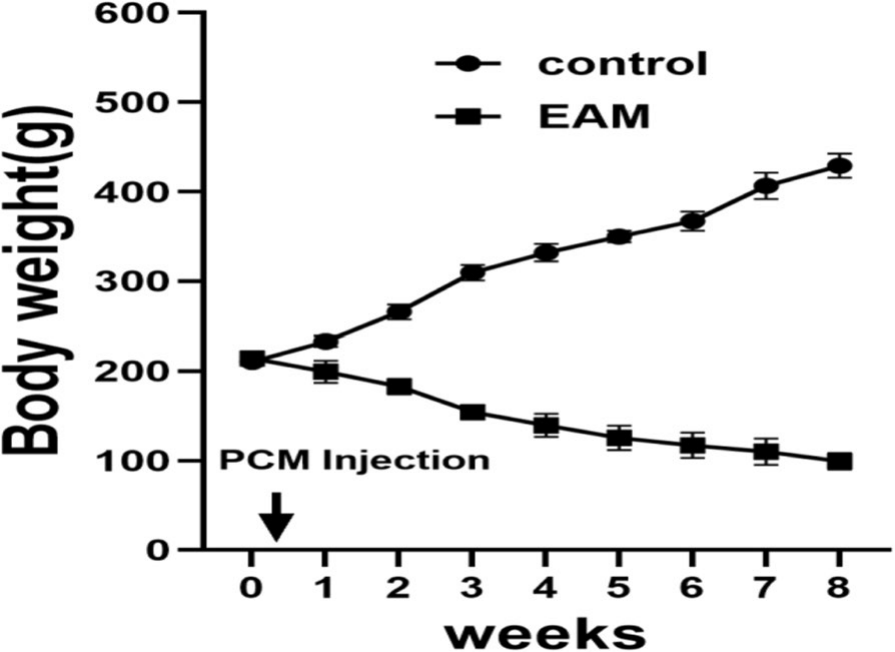
The body weight change of rats was monitored within 8 weeks post infection (*n* = 5 per group). The body weight of EAM rats gradually decreased after PCM immunization, and the body weight decreased significantly around 3–4 weeks

Under baseline conditions at 8–9 weeks of age, there were no significant differences between control group rats and experimental group rats under normal conditions. From Day 1 we observed the activity, diet and mental situation in each group of rats and tested weight every 3 days. In each group, all rats were still alive on Day 56. Eight days after the first immunization, the EAM group rats were depressed, had decreased activity, dark colour, and a reduced diet. Moreover, after immunization with PCM, the body weight of EAM rats showed a progressive decrease,and the body weight decreased significantly approximately 3–4 weeks (Fig. 1).

### EAM induced acute HF and chronic HF

**Fig. 2:**
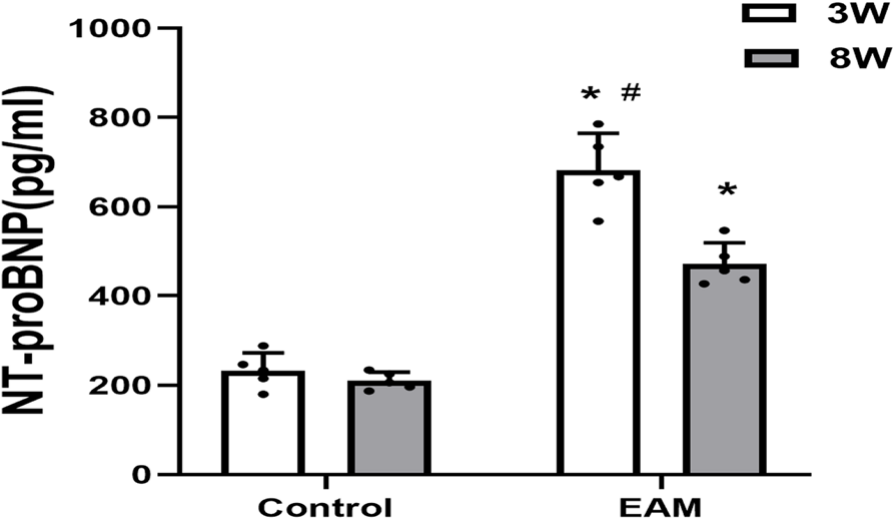
Serum NT-proBNP concentration was detected by ELISA. (*P < 0.05 as compared with control group, #*P* < 0.05 as compared with EAM group (8 week))

LVEDd, LVEDs, EF and FS are the main indicators of cardiac function detected by echocardiographic examination. Echocardiography was performed on Day 21 and 56 in PCM-immunized rats, and the results showed that LVEDd and LVEDs were significantly increased in the EAM group compared to the control group at 3 weeks and 8 weeks. However, in comparison with control rats, PCM-immunized rats had significantly lower EF and FS on Day 21 and 56 of immunization (Table 2). Additionally, serum NT-proBNP concentrations were significantly higher in EAM group rats than in control group rats, both at 3 weeks and 8 weeks after immunization. Not surprisingly, the serum NT-proBNP concentrationin the EAM group (3 weeks) was higher than that in the EAM group (8 weeks), indicating that acute HF was induced during the acute peak of inflammation (Fig. 2). In our study, the incidence of HF induced by EAM was 100%. In summary, myocarditis predisposes patients to decreased cardiac function and is both a common and important cause of acute and chronic HF.

**Table 2:** Evaluation of heart function in each group by echocardiography

Group	LVEDd(mm)	LVEDs(mm)	EF(%)	FS(%)
**Day 21**				
**Control group**	5.64 ± 0.29	2.46 ± 0.11	85.81 ± 1.51	56.25 ± 2.21
**EAM group**	6.37 ± 0.35^*^	4.32 ± 0.29^*^	63.98 ± 4.03^*^	35.92 ± 2.99^*^
**Day 56**				
**Control group**	5.68 ± 0.45	2.56 ± 0.34	86.88 ± 3.33	57.83 ± 4.41
**EAM group**	6.30 ± 0.30^*△^	3.74 ± 0.32^*△^	70.70 ± 3.82^*#^	42.15 ± 4.52^*#^

^*^P < 0.05 vs Control group

^#^*P* < 0.05 vs EAM group(3W)

^△^*P* > 0.05 vs EAM group(3 W)

### Levels of inflammation and fibrosis in EAM-induced HF rats

**Fig. 3:**
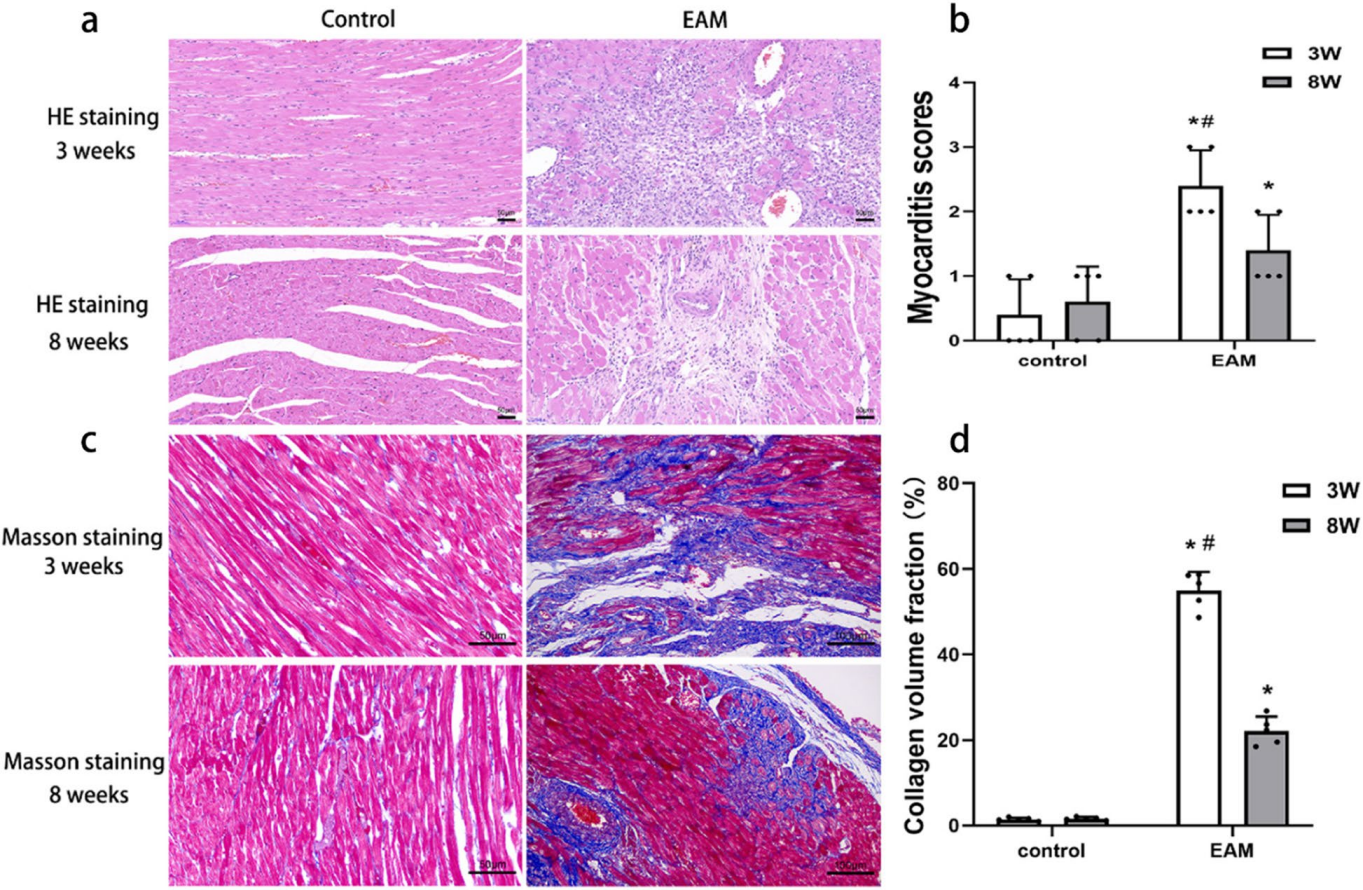
HE staining and masson staining to assess myocardial tissue inflammatory infiltration and fibrosis. **a** Representative photomicrograph of HE staining; **b** The myocarditis scores for each group; **c** Representative photomicrograph of masson staining; **d** The collagen volume fraction for each group (*P < 0.05 as compared with corresponding control group, #*P* < 0.05 as compared with EAM group(8 weeks))

Compared with the normal control group and EAM group (8 weeks), the EAM group (3 weeks) showed obvious inflammatory infiltration in HE staining and obvious myocardial fibrosis by Masson’s trichrome staining. Moreover, inflammatory infiltration and myocardial fibrosis were significantly higher in the EAM group (8 weeks) compared to EAM group (3 weeks) than in the corresponding control group (Fig. 3).

### Serum ET-1 levels in EAM-induced HF rats

**Fig. 4:**
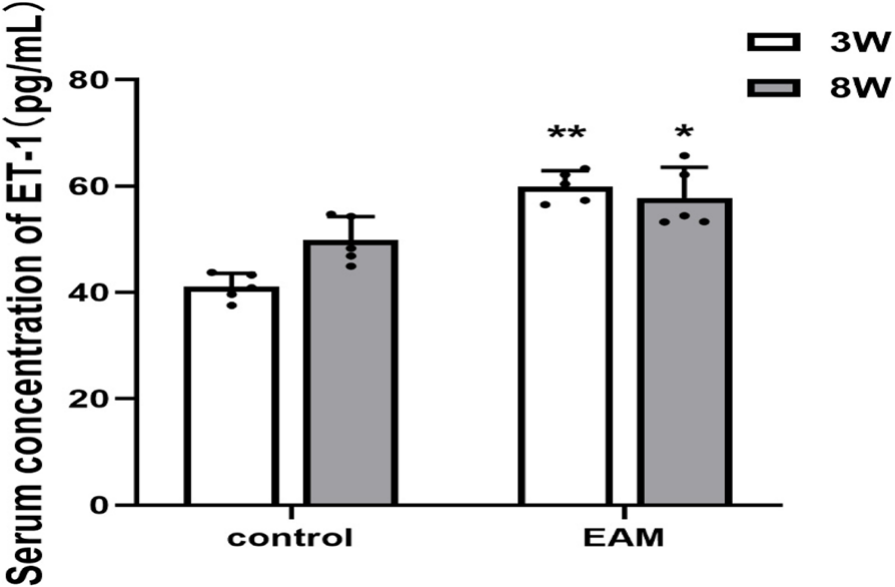
Serum concentration of ET-1 measured by ELISA. (**P < 0.01 as compared with control group (3 weeks),*P < 0.05 as compared withcontrol group(8 weeks))

We used ELISA to detect the expression level of ET-1 in EAM-induced HF rats. The results showed that the serum ET-1 concentrations were significantly higher in both acute and chronic HF rats than in the corresponding control group (Fig. 4).

### Localization and expression of ETAR and ETBR in the hearts of EAM rats

**Fig. 5:**
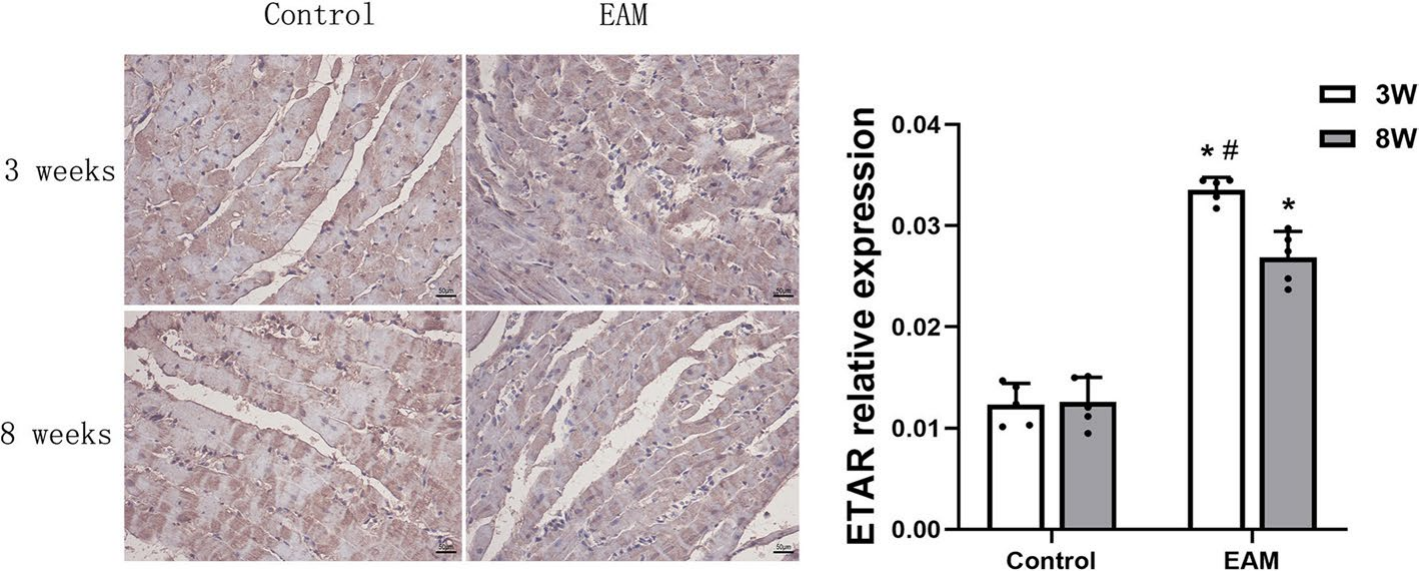
Micrography of ETAR protein in myocardial tissue detected by immunohistochemistry assay.( *P < 0.05 as compared with corresponding control group, #*P* < 0.05 as compared with EAM group (8 weeks))

**Fig. 6:**
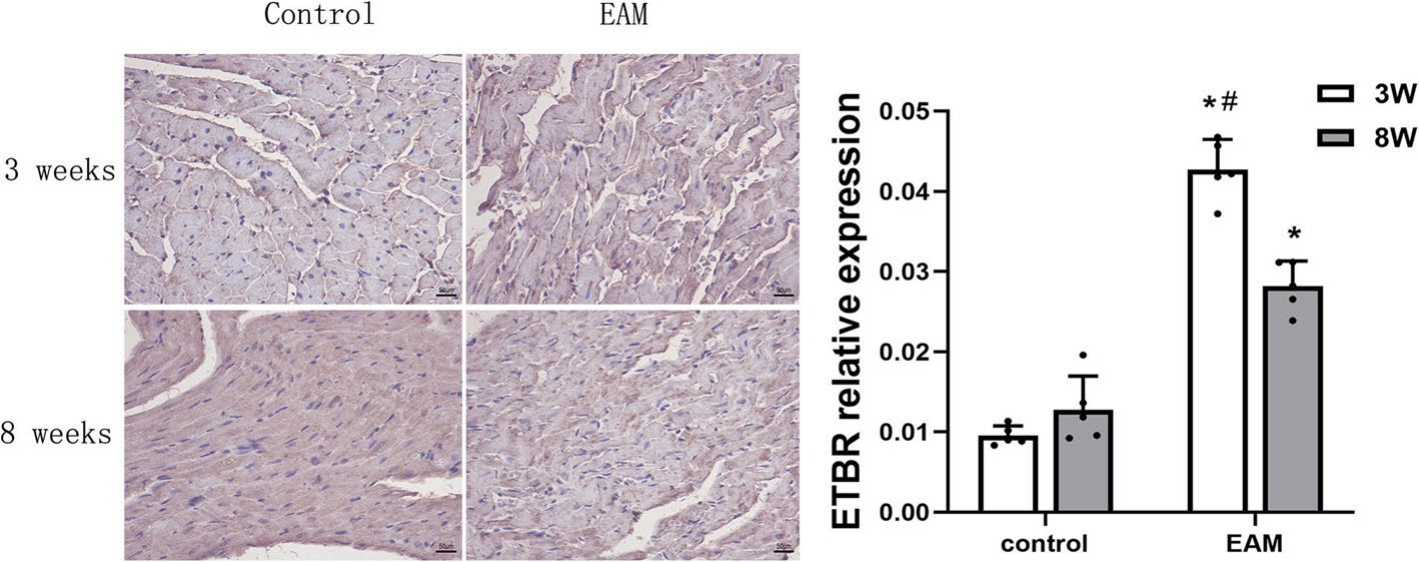
Micrography of ETBR protein in myocardial tissue detected by immunohistochemical method. ( *P < 0.05 as compared with corresponding control group, #*P* < 0.05 as compared with EAM group (8 weeks))

To investigate the relationship between ETAR and ETBR and HF, we first reviewed and collected paraffin sections of myocardial tissues from each group of rats, followed by immunohistochemical methods to detect the expression of ETAR and ETBR in heart tissues. The immunohistochemistry results demonstrated that ETAR and ETBR were primarily localized in the endochylema. The immunostaining of ETAR and ETBR was performed by greyscale test with a computer-assisted image analysis system and the results showed that the levels of ETAR and ETBR were significantly increased in the EAM group (3 weeks) and EAM group (8 weeks). However, there were no significant differences between the control groups at 3 weeks and 8 weeks (Figs. 5 and 6). These data indicated that both ETAR and ETBR were expressed in the hearts of rats with EAM-induced acute and chronic HF, and both were located in the endochylema.

### Expression of ETAR and ETBR in myocardial tissue of EAM-induced HF rats

**Fig. 7:**
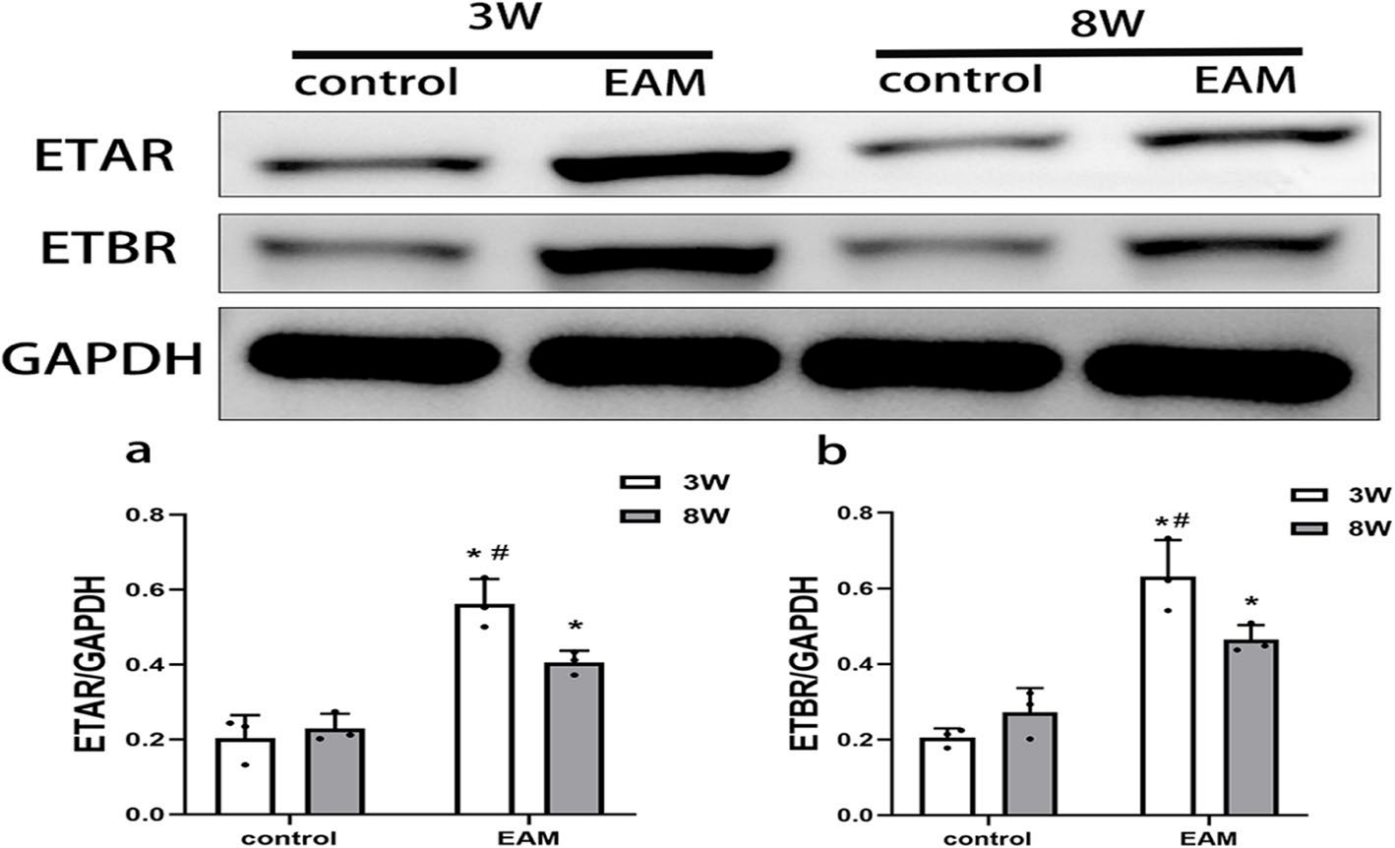
The expression levels of ETAR and ETBR in myocardial tissue were detected by western blot. The expression of ETAR and ETBR is significantly increased in EAM group (3 weeks) and EAM group (8 weeks) in vivo. (* *P* < 0.05 as compared with correponding control group, #*P* < 0.05 as compared with EAM group (8 weeks))

We used western blotting to detect the protein expression levels of ETAR and ETBR in myocardial tissues. The expression of ETAR and ETBR was significantly higher in both the EAM-induced acute HF and chronic HF groups than in the corresponding control group (Fig. 7). Meanwhile, the expression levels of ETAR and ETBR were significantly higher in the acute HF group than in the chronic HF group, suggesting that ETAR and ETBR may play an important role in EAM-induced acute and chronic HF, especially in acute HF.

### ETAR and ETBR protein expression levels were elevated in a myocardial inflammatory injury model in vitro

**Fig. 8:**
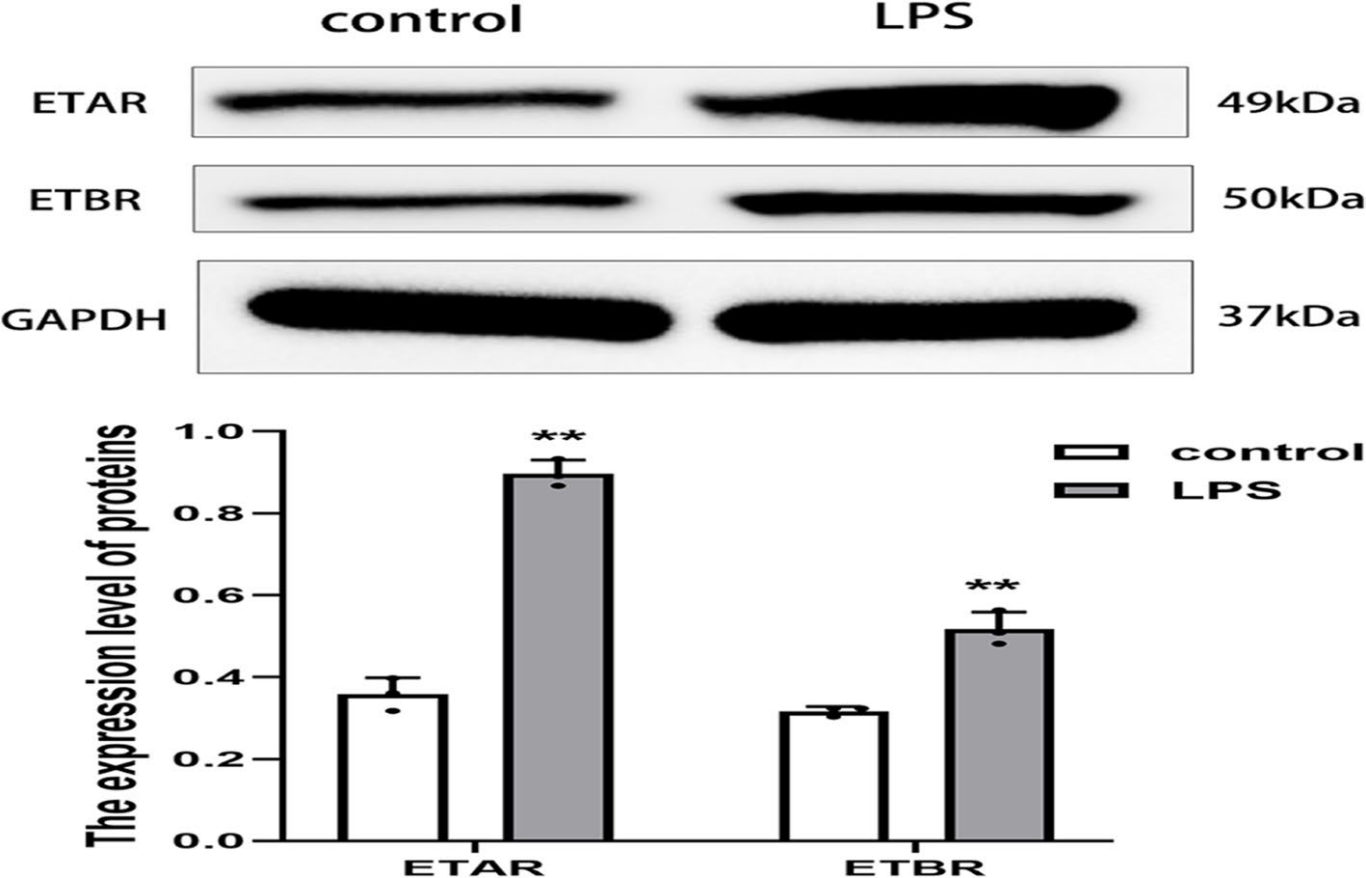
The expression of ETAR and ETBR proteins in myocardial inflammatory injury model in vitro (** *P* < 0.01 as compared with corresponding control group)

To explore the role of ETAR and ETBR activation in heart failure induced by EAM, we stimulated H_9_C_2_ rat cardiomyocytes with LPS to construct a myocardial inflammatory injury model in vitro. It was found that ETAR and ETBR protein levels were significantly increased in H_9_C_2_ cells stimulated by LPS (Fig. 8).

### Identification of the transfection effect of the ETAR and ETBR knockdown plasmids

**Fig. 9:**
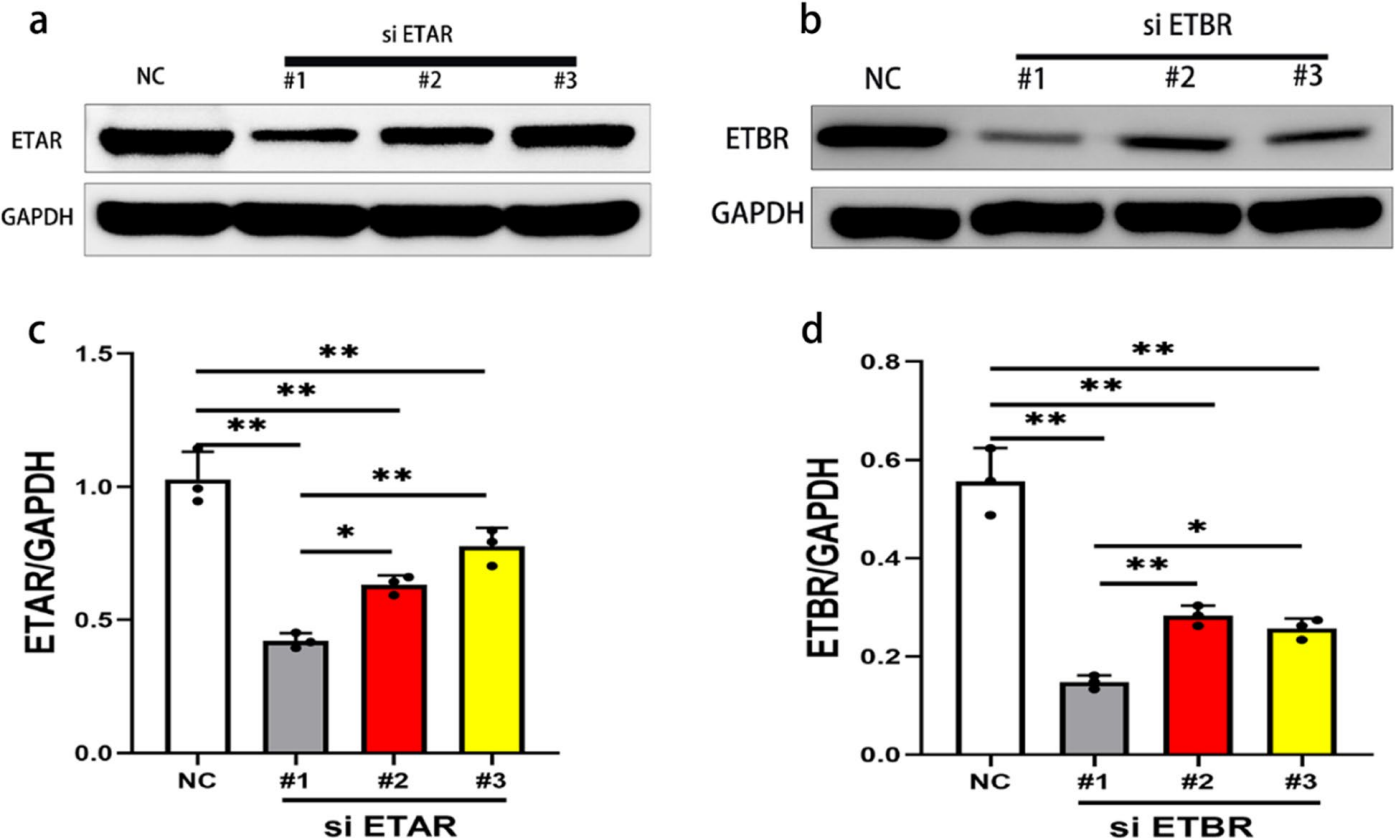
Identification of the transfection effect of the ETAR and ETBR knockdown plasmids. **a** and **c** The expression of ETAR protein in cells of the siRNA-1 group was significantly lower than in the groups of siRNA-2, siRNA-3, and the negative control (NC) group (*P < 0.05,**P < 0.01)). **b** and **d** The expression of ETBR protein in cells of the siRNA-1 group was significantly lower than in the groups of siRNA-2, siRNA-3, and the negative control (NC) group (*P < 0.05,**P < 0.01))

Western blot results showed that the expression of ETAR protein in H_9_C_2_ cells in the si ETAR-1 group was significantly lower than that in the si ETAR-2 group, si ETAR -3 group and normal control group (Fig. 9a and c). The protein expression of ETBR in H_9_C_2_ cells in the si ETBR-1 group was significantly lower than that in the si ETBR-2 group, si ETBR-3 group and normal control group (Fig. 9b and d). Therefore, si ETAR-1 and si ETBR-1 had the best interference effect, so they were selected as the interference fragment for further experiments.

### ETAR knockdown decreased inflammatory cytokine TNF-α and IL-1β levels while ETBR knockdown increased TNF-α and IL-1β levels

**Fig. 10:**
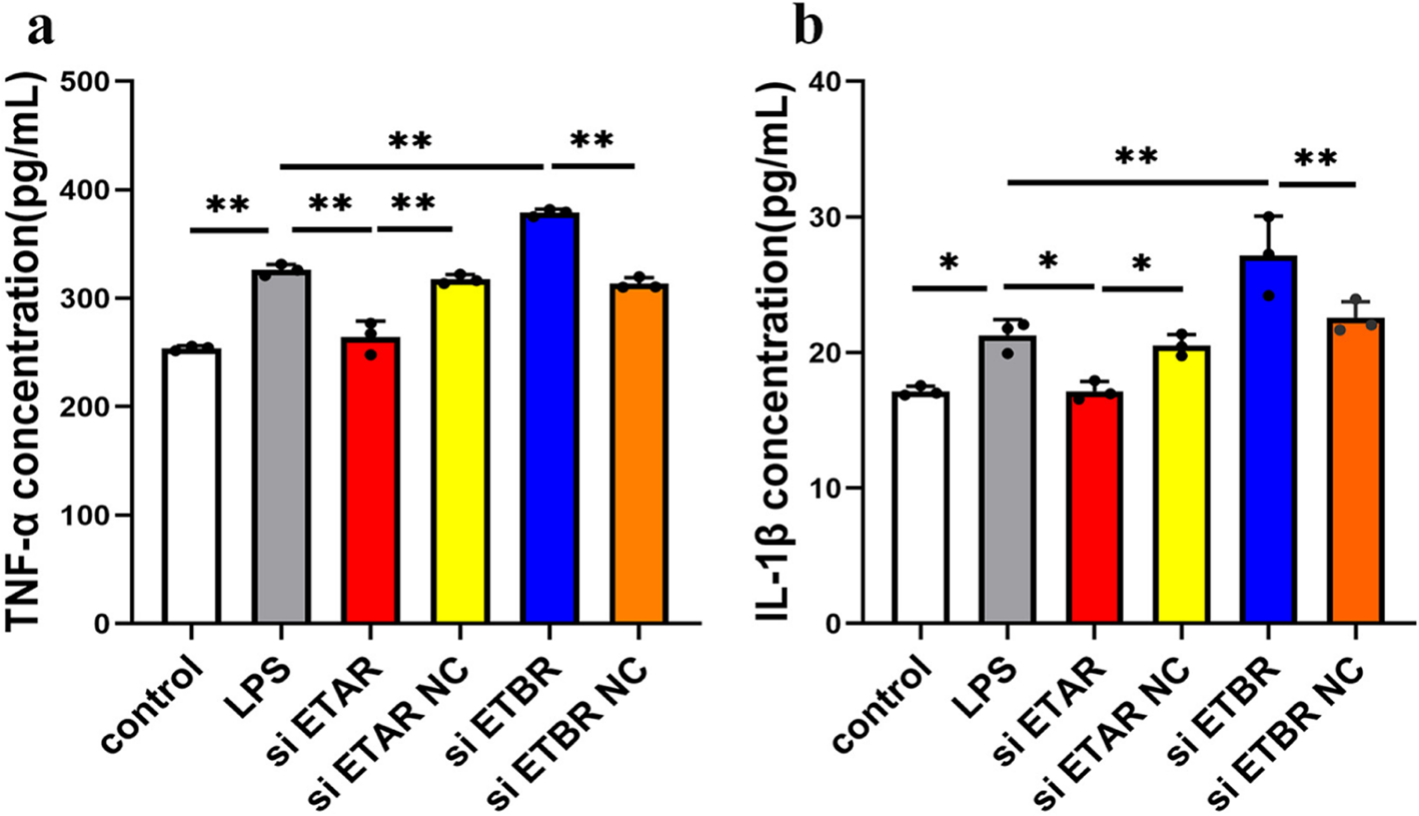
The change of inflammatory cytokine TNF-α and IL-1β levels after ETAR and ETBR knockdown in myocardial inflammatory injury model in vitrol. **a** The concentration of inflammatory cytokine TNF-α was detected by ELISA (**P < 0.01). **b** The concentration of inflammatory cytokine IL-1β was detected by ELISA ( *P < 0.05,**P < 0.01)

H_9_C_2_ cells were transfected with siRNAs targeting ETAR and ETBR to investigate the levels of inflammatory factors after ETAR and ETBR knockdown. As a result, the concentrations of the inflammatory cytokines TNF-α and IL-1β in the H_9_C_2_ cell supernatant were significantly increased in the myocardial inflammatory injury model. Moreover, the results revealed that the levels of the inflammatory cytokines TNF-α and IL-1β were significantly reduced after ETAR knockdown, while the inflammatory cytokines TNF-α and IL-1β were significantly increased after ETBR knockdown (Fig. 10a and b). Therefore, ETBR activation contributed to alleviating myocardial inflammation while ETAR activation may exacerbate myocardial inflammation.

## Discussion

HF is a clinical syndrome caused by structural and/or functional abnormalities of the heart, often manifesting as progressive and severe diastolic dysfunction or severe systolic dysfunction (defined as EF < 50%) [19–21]. To date, HF remains the leading cause of mortality, hospitalization, and poor quality of life worldwide [22].

There are many causes of HF, such as hypertension, type 2 diabetes, coronary artery disease, and valvular heart disease, most of which contribute to the development of HF by affecting the heart and blood vessels [23]. However, there is a lack of attention to the relationship between myocarditis and HF. Myocardial inflammation may also persist in both the acute and chronic phases of myocarditis, and persistent inflammation is characterized by continued myocardial cell injury and loss of cardiac cells, ultimately leading to fibrosis and nonischemic HF [24]. We constructed an EAM model by PCM. Myocardial tissue inflammatory cell infiltration and myocardial fibrosis were significantly higher in the EAM group (3 weeks) and EAM group (8 weeks) than in the control group, confirming that inflammation persisted in both the acute and chronic HF induced by EAM. After PCM immunization, the rats began to show signs of depression, decreased activity, reduced diet and weight loss. We further assessed the cardiac function of the rats in each group at 3 weeks and 8 weeks by echocardiography. Both in the EAM group (3 weeks) and in the EAM group (8 weeks), the rats showed significant changes in cardiac structure and function, mainly in the form of enlarged left ventricles and reduced left ventricular EF. Moreover, compared with that in the control group, the serum NT-proBNP concentration in the EAM group (both 3 weeks and 8 weeks) was significantly increased after immunization, confirming that EAM led to the development of nonischemic HF. Therefore, over time, myocarditis can induce acute HF and chronic HF and is an important cause of HF.

Because HF usually leads to a poor prognosis, the pathophysiological mechanisms of HF have been continuously studied. Endothelial dysfunction is a newly discovered phenomenon that usually coincides with chronic HF [9]. Therefore, in recent years, an increasing number of studies have begun to investigate the role of endothelial dysfunction in HF. Previous studies have shown that ET-1, in addition to being a potent vasoconstrictor, may induce endothelial dysfunction by decreasing NO bioavailability and is one of the biomarkers of endothelial dysfunction [11]. Therefore, we clarified the relationship between endothelial dysfunction and HF induced by EAM by detecting ET-1 levels in EAM rats. Our study found that compared to controls, the concentration of ET-1 was elevated in rats with acute HF and chronic HF induced by EAM, which is consistent with reports from previous studies [25–27]. Therefore, endothelial dysfunction may play a crucial role in the development and progression of HF, especially EAM-induced HF.

ET-1 acts by activating two receptor subtypes, ETAR and ETBR, depending on the cell type. ETBR on endothelial cells causes vasodilation by inducing NO release, whereas ETAR and ETBR located on smooth muscle cells and fibroblasts trigger vasoconstriction, inflammation and fibrosis [28]. ET-1 receptor antagonists are currently used clinically in the treatment of patients with pulmonary artery hypertension [29]. However, the role of ET-1 receptors in HF is still controversial, and no previous studies have reported the role of ETR in HF induced by EAM. Our study found that, similar to ET-1, the expression levels of ETAR and ETBR were significantly higher in the myocardial tissue of both acute HF and chronic HF rats than in control rats. Therefore, we suggest that activation of ETAR and ETBR plays an important role in EAM-induced HF. Although the role of ETBR in the pathophysiology and remodelling of HF has not been clearly defined [30], a large number of studies have at least demonstrated that dual ETAR/ETBR antagonists are beneficial for HF. In short-term (2 weeks) therapy, the dual ETAR/ETBR antagonist bosentan (1000 mg twice daily) has been shown to produce immediate and sustained haemodynamic benefit in patients with HF [31]. Moreover, Valero-Munoz et al. (2016) demonstrated that dual ETAR/ETBR inhibition abrogates unfavourable cardiac remodelling through anti-cardiomyocyte hypertrophic mechanisms and reduces stiffness, thereby improving HFpEF [32].

To further explore the role of ETAR and ETBR in EAM-induced HF, we constructed a myocardial inflammatory injury model in vitro by LPS stimulation in H_9_C_2_ cells. The results revealed that the levels of the inflammatory cytokines TNF-α and IL-1β increased significantly after LPS stimulation of H_9_C_2_ cardiomyocytes, suggesting that the myocardial inflammatory injury model was successfully constructed. Moreover, consistent with the results of the experiments in vivo, the protein expression levels of ETAR and ETBR were significantly elevated in the myocardial inflammatory injury model in vitro. Subsequently, we induced ERAR and ETBR gene silencing by RNA interference technology. The results showed that ETAR gene silencing ameliorated LPS-induced cardiomyocyte inflammation, while ETBR gene silencing exacerbated LPS-induced cardiomyocyte inflammation. Therefore, ETAR and ETBR may play a role in EAM-induced HF by regulating cardiomyocyte inflammation, which explains why the expression of ETAR and ETBR in myocardial tissues was lower in the EAM group (8 weeks) than in the EAM group (3 weeks).

Regarding the diagnosis of HF, the 2016 European Society of Cardiology (ESC) guidelines suggest that BNP, although it can be used to exclude HF, is not diagnostic because of its low positive predictive value [33]. Moreover, BNP is influenced by many factors, such as age, sex, obesity and higher heart rate in atrial fibrillation [34]. For these reasons, many other biomarkers of HF have started to emerge, such as galectin-3, growth differentiation factor-15 (GDF-15), and mid-regional pro-adrenomedullin [35]. From our findings, it appears that the ETAR and ETBR have at least the potential to act as potent biomarkers of HF induced by myocarditis.

## Conclusion

Our study found that the levels of ET-1, ETAR, and ETBR were significantly elevated in EAM-induced acute and chronic heart failure. Moreover, ETAR gene silencing contributed to alleviating myocardial inflammation, while ETBR gene silencing exacerbated myocardial inflammation, suggesting that ETAR and ETBR can be regarded as novel biomarkers to characterize heart failure induced by EAM.

### Supplementary Information

**Additional file 1: Figure 1.** The original gel and the whole membrane of ETAR and ETBR in myocardial tissue were detected by western blot (Corresponding to Fig. 7 in the manuscript). **Figure 2.** The original gel and the whole membrane of ETAR and ETBR proteins expression in myocardial inflammatory injury model in vitro (Corresponding to Fig. 8 in the manuscript). **Figure 3.** Identification of the transfection effect of the ETAR knockdown plasmids (Corresponding to Fig9a in the manuscript). **Figure 4.** Identification of the transfection effect of ETBR knockdown plasmids (Corresponding to Fig. 9b in the manuscript).

## Data Availability

The datasets used and/or analyzed during the current study are available from the corresponding author on reasonable request.
