# Supplementary material for: Activation of ETAR and ETBR in myocardial tissue characterizes heart failure induced by experimental autoimmune myocarditis
Source: BMC Cardiovasc Disord. 2024 Jan 2;24:11. doi: 10.1186/s12872-023-03658-1 (PMC10763419; doi:10.1186/s12872-023-03658-1)
Supplement: Supplementary file 1 — Additional file 1: Figure 1. The original gel and the whole membrane of ETAR and ETBR in myocardial tissue were detected by western blot (Corresponding to Fig. 7 in the manuscript). Figure 2. The original gel and the whole membrane of ETAR and ETBR proteins expression in myocardial inflammatory injury model in vitro (Corresponding to Fig. 8 in the manuscript). Figure 3. Identification of the transfection effect of the ETAR knockdown plasmids (Corresponding to Fig9a in the manuscript). Figure 4. Identification of the transfection effect of ETBR knockdown plasmids (Corresponding to Fig. 9b in the manuscript). [file 12872_2023_3658_MOESM1_ESM.pdf]

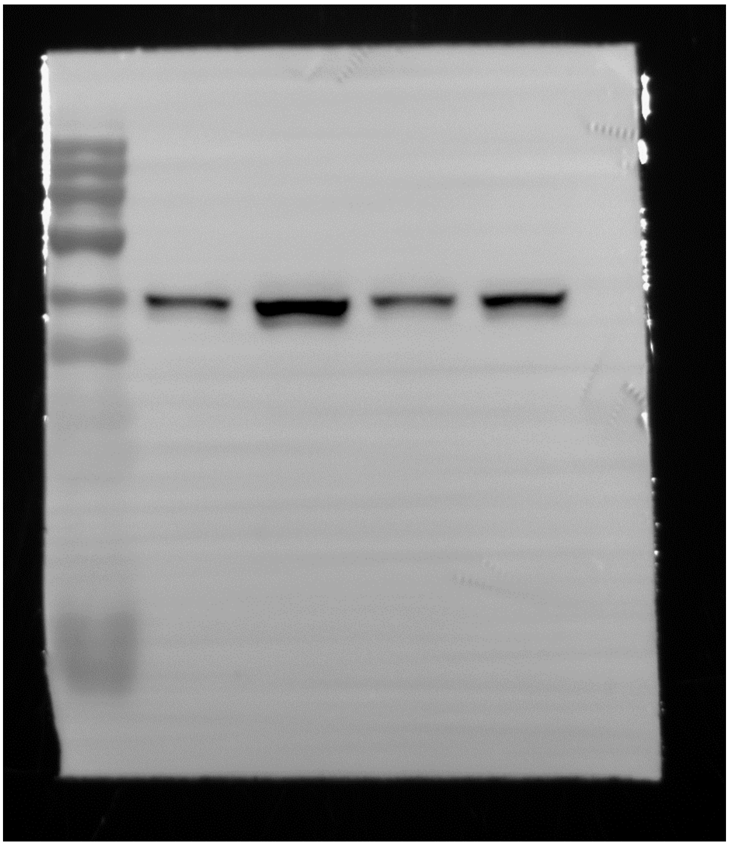

Figure 1． 1ETBR

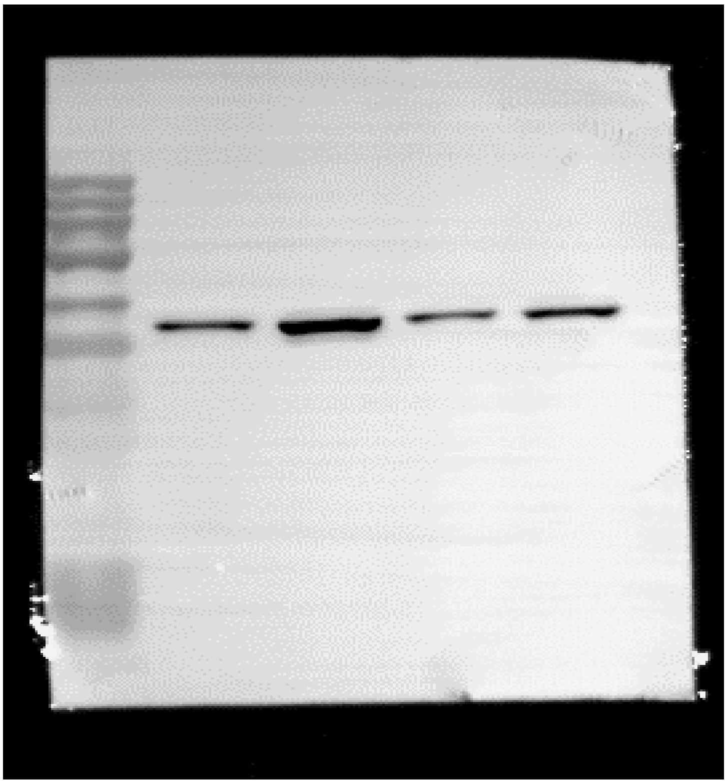

Figure 1.2ETAR

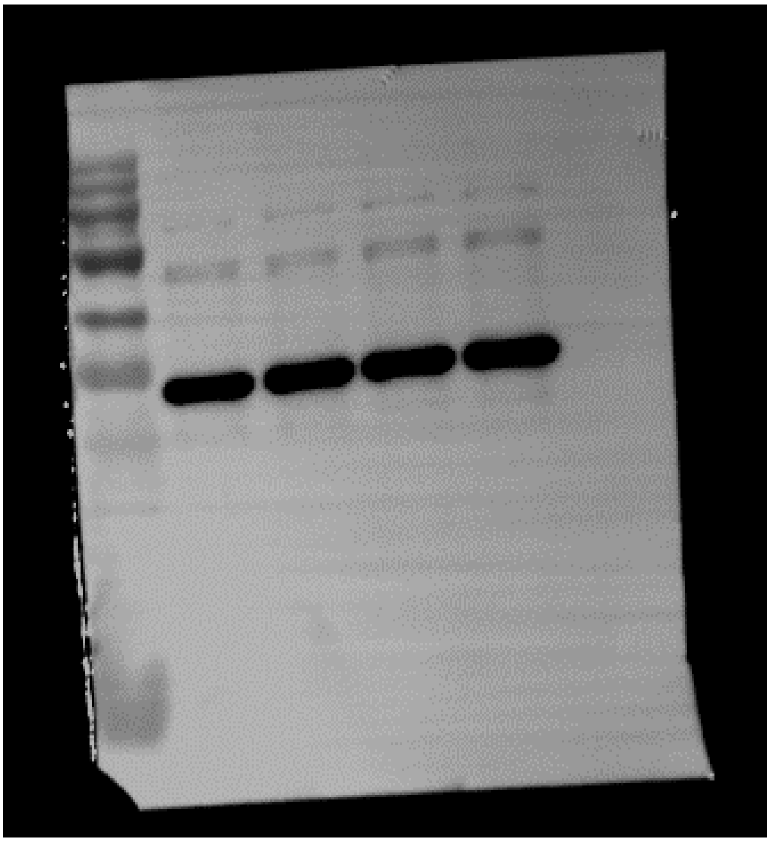

Figure 1.3 GAPDH

Figure 1 The original gel and the whole membrane of ETAR and ETBR in myocardial tissue were detected by western blot(Corresponding to Figure 7 in the manuscript).

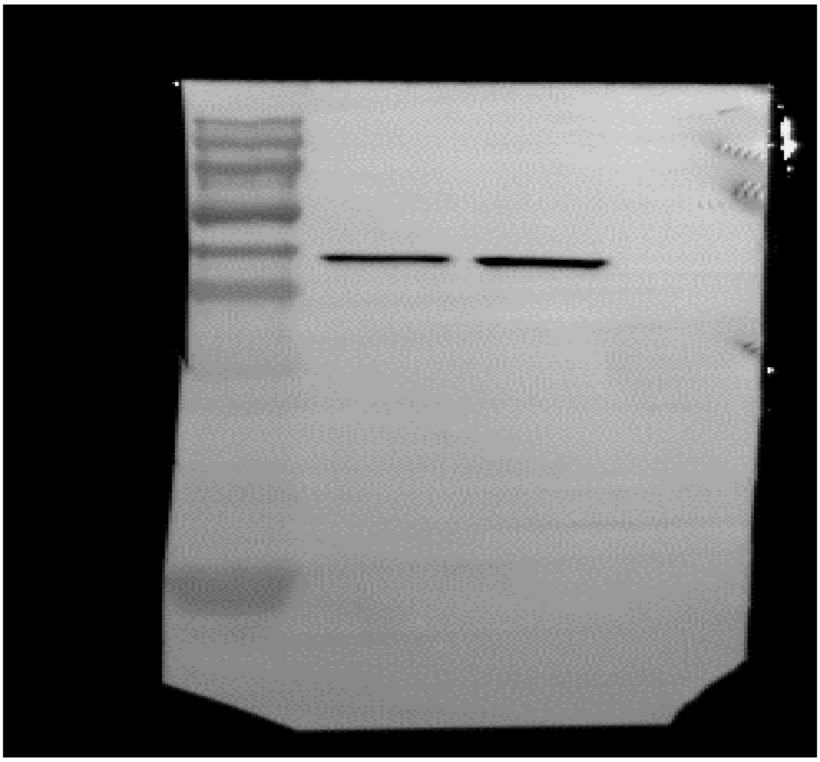

Figure 2.1ETBR

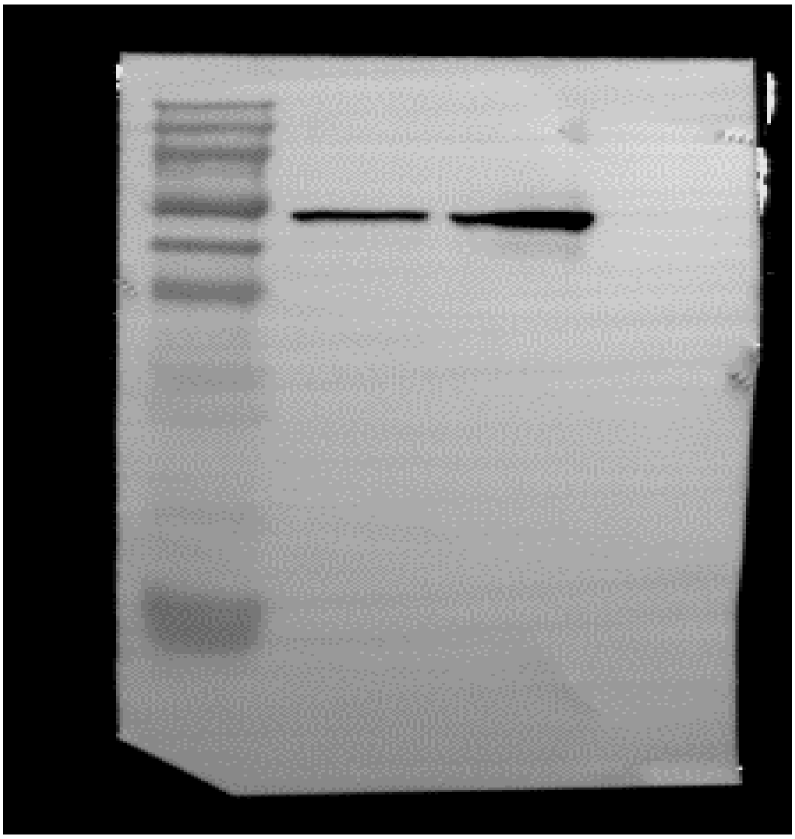

Figure 2.2 ETAR

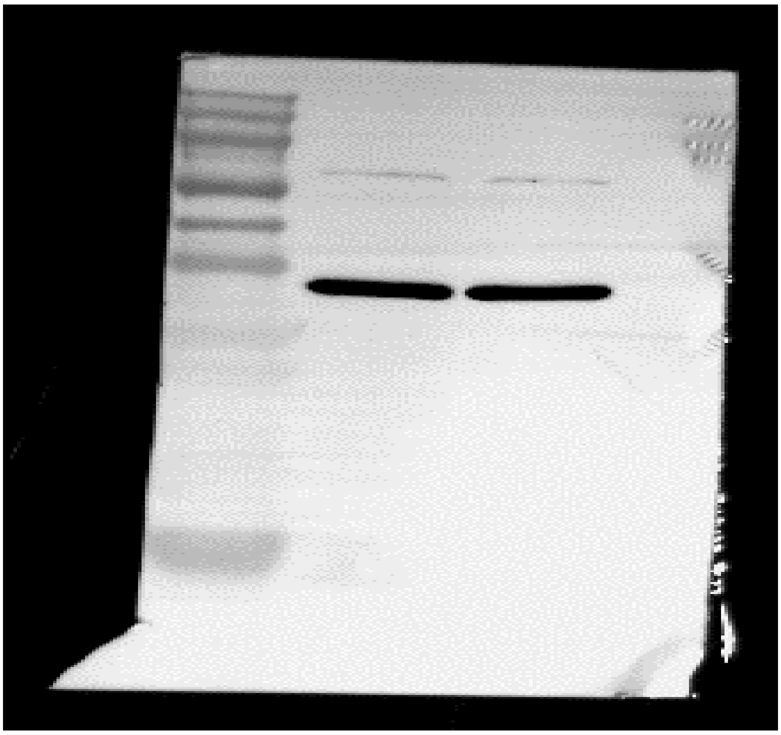

Figure 2.3GAPDH

Figure 2 The original gel and the whole membrane of ETAR and ETBR proteins expression in myocardial inflammatory injury model in vitro(Corresponding to Figure 8 in the manuscript)

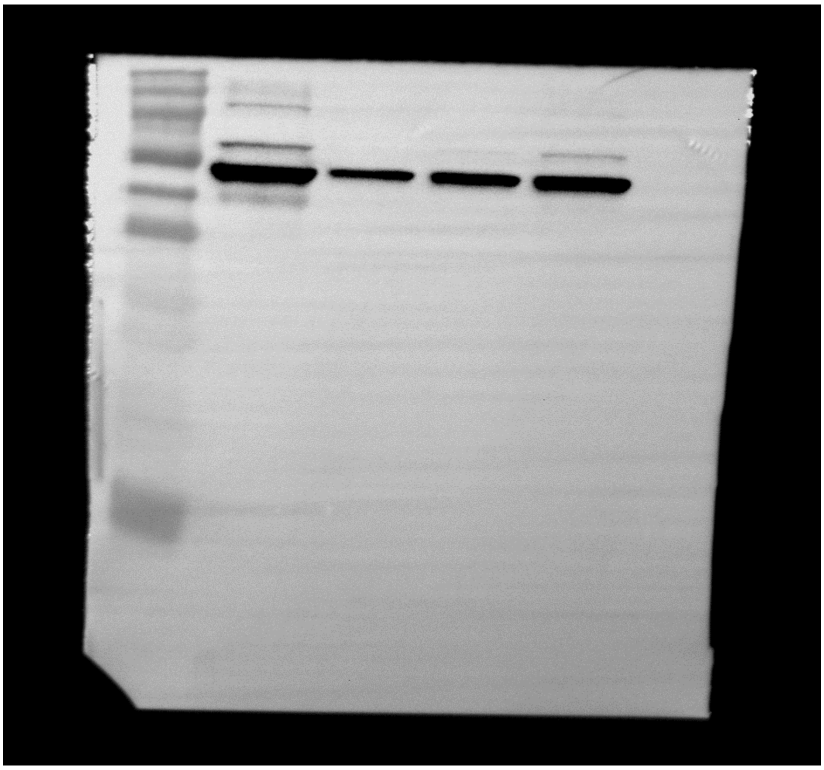

Figure 3.1ETAR

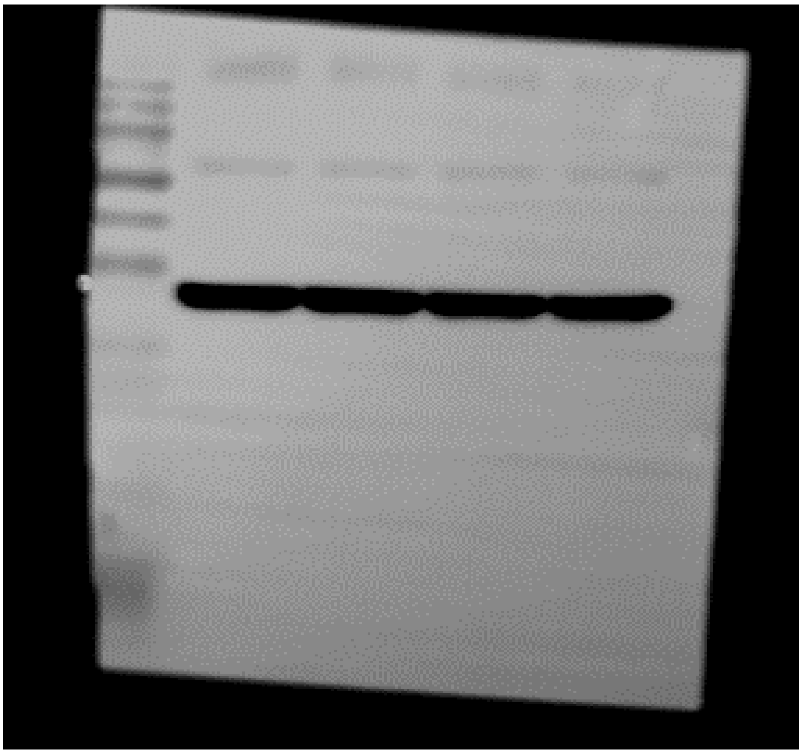

Figure3.2GAPDH

Figure 3 Identification of the transfection effect of the ETAR knockdown plasmids(Corresponding to Fig9a in the manuscript).

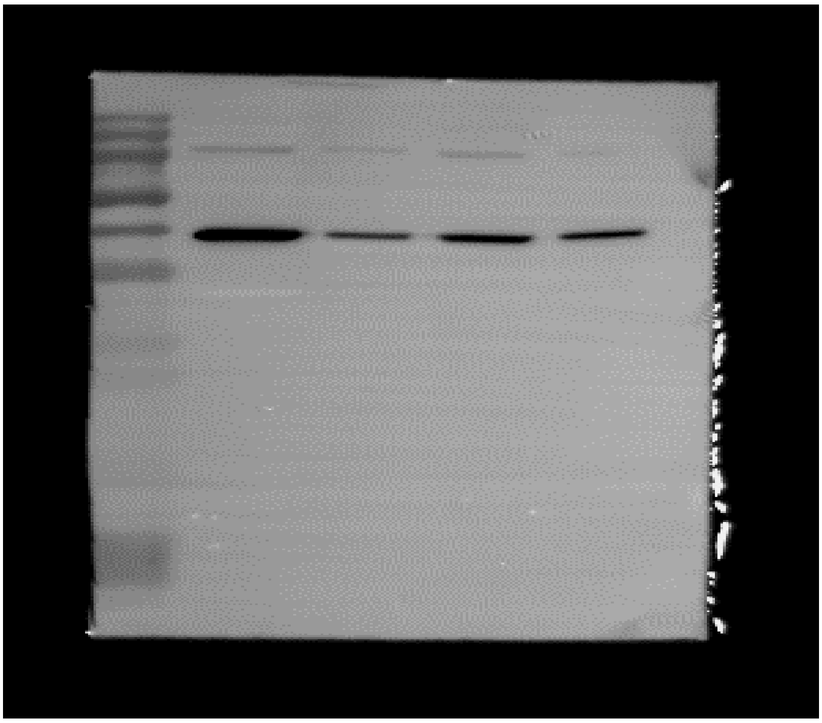

Figure 4.1ETBR

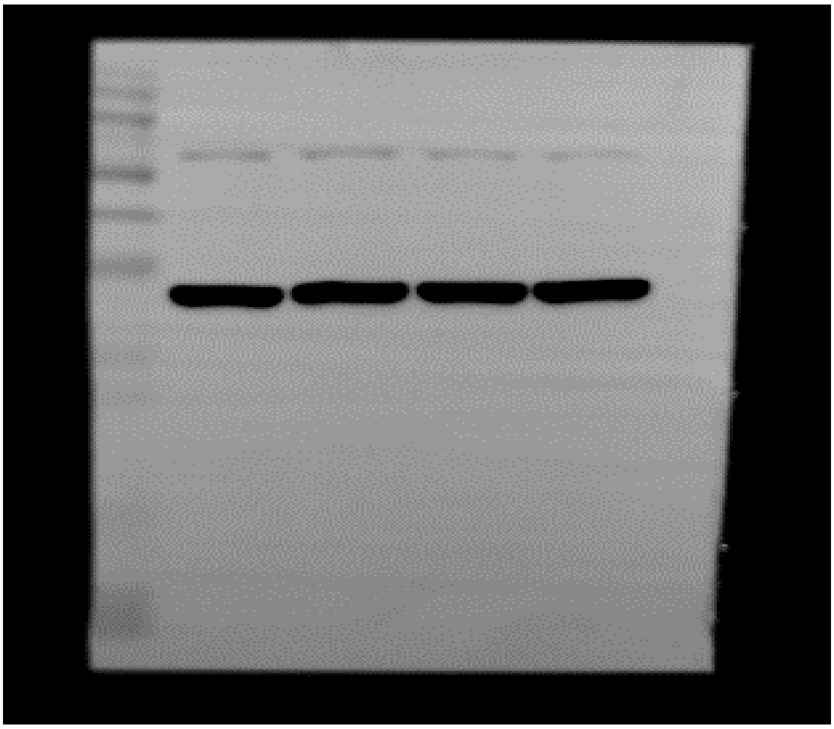

Figure 4.2GAPDH

Figure 4 Identification of the transfection effect of ETBR knockdown plasmids(Corresponding to Fig9b in the manuscript).
